# A Forecasting Model Based on High-Order Fluctuation Trends and Information Entropy

**DOI:** 10.3390/e20090669

**Published:** 2018-09-04

**Authors:** Hongjun Guan, Zongli Dai, Shuang Guan, Aiwu Zhao

**Affiliations:** 1 School of Management Science and Engineering, Shandong University of Finance and Economics, Jinan 250014, China; 2 Rensselaer Polytechnic Institute, Troy, NY 12180, USA; 3 School of Management, Jiangsu University, Zhenjiang 212013, China

**Keywords:** high-order fluctuation trends, forecasting, information entropy, neutrosophic sets

## Abstract

Most existing high-order prediction models abstract logical rules that are based on historical discrete states without considering historical inconsistency and fluctuation trends. In fact, these two characteristics are important for describing historical fluctuations. This paper proposes a model based on logical rules abstracted from historical dynamic fluctuation trends and the corresponding inconsistencies. In the logical rule training stage, the dynamic trend states of up and down are mapped to the two dimensions of truth-membership and false-membership of neutrosophic sets, respectively. Meanwhile, information entropy is employed to quantify the inconsistency of a period of history, which is mapped to the indeterminercy-membership of the neutrosophic sets. In the forecasting stage, the similarities among the neutrosophic sets are employed to locate the most similar left side of the logical relationship. Therefore, the two characteristics of the fluctuation trends and inconsistency assist with the future forecasting. The proposed model extends existing high-order fuzzy logical relationships (FLRs) to neutrosophic logical relationships (NLRs). When compared with traditional discrete high-order FLRs, the proposed NLRs have higher generality and handle the problem caused by the lack of rules. The proposed method is then implemented to forecast Taiwan Stock Exchange Capitalization Weighted Stock Index and Heng Seng Index. The experimental conclusions indicate that the model has stable prediction ability for different data sets. Simultaneously, comparing the prediction error with other approaches also proves that the model has outstanding prediction accuracy and universality.

## 1. Introduction

For stock market forecasts, summarizing the rules that can be used for future predictions from historical data is crucial. At the same time, due to the noise that is contained in the actual data, Song and Chissom [1,2,3] proposed a fuzzy time series method for general rule extraction. On this basis, some scholars proposed first-order models [4,5,6], whereas others further studied high-order models [7,8]. These studies not only imply that high-order models can reflect historical trends, but also emphasize the importance of the relationship between historical trends and current state.

However, most of above models summarize the logical relationships between current and historical trends that are only based on discrete states. In fact, in addition to the simple state-to-state relationship, the relationships between the historical states and the current states are also related to other features. For example, Lee et al. [9] suggested that the fluctuation trends in related stock markets are linked with each other. From this point of view, Lee et al. proposed a prediction model that is based on two related stock markets that extended such models to two-factor high-order prediction models. In addition to the range of external influences, other internal features of a time series are ignored by traditional autoregressive models. For example, a typical phenomenon, called “callback”, occurs after a period of continuous rise, which means that the inconsistencies in historical fluctuation trends might have considerable impacts on future fluctuation trends. In this area, Zhang et al. [10] extracted the inconsistent features of different types of digital signals and selected the optimal feature subsets from 16 entropy features. Jiang et al. [11] proposed a feature fusion model that is based on information entropy (IE) and probabilistic neural network, which uses IE theory to extract the characteristic entropies in vibration signals. These studies inspired us to think that IE can be employed to describe the inconsistency in a period of fluctuation in a time series.

The concept of information entropy was first proposed by Shannon in 1948 [12]. IE is a tool for measuring the degree of ordering of systems. The lower the IE, the less chaos, and the higher the order. Conversely, the larger the IE, the lower the order. IE solves the problem of quantification and describing system complexity and inconsistency. For example, in order to indicate the inconsistency of a battery due to small deviations and uncertainties in its production process, Duan et al. [13] developed a comprehensive evaluation method for battery inconsistency based on IE. Xu et al. [14] proposed the information entropy risk measure, which applied IE to describe the uncertainty in decision risk and the inconsistency of decision information. Based on the uncertainty of rainfall distribution and the inconsistency of information, Wang et al. [15] employed IE to analyze the time-variation in similar-scale rainfall networks in cities. Information entropy has been widely used in many other situations, such as graphics detection [16], algorithm optimization [17], environmental evaluation [18], and big data analysis and mining [19].

In order to fully express the three states of up, down, and inconsistency of a period in history in a logical relationship and to conveniently compare them, the expression variable should have three dimensions. This is similar to the structure of neutrosophic sets (NSs) and the similarity comparisons of different NSs. The NS was introduced by Smarandache [20], which consists of true membership, indeterminacy membership, and false membership. From the perspective of information representation, scholars have proposed three specific concepts that are based on the neutrosophic set: single-valued NSs [21], interval-valued NSs [22], and simplified NSs [23], including single-valued and interval-valued NSs [24]. These concepts seek a more detailed representation of the information, enabling the NSs to express inconsistent information more accurately. At present, NSs have attracted the attention of scholars in various fields. For instance, Abdel-Basse et al. [25] discussed the role of NSs in urban construction and further analyzed its advantages in the construction of imperfect and incomplete information systems. Şahin et al. [26] discussed the application of single-valued NSs in decision making. Van et al. [27] applied NSs to green supplier evaluation and selection. In addition, since similarity is an important method for measuring NSs, similarity was also used in numerous studies [28,29,30].

As mentioned above, stock market fluctuation is a dynamic nonlinear system. A common question is how complex systems can be synchronized and which parameters have a direct impact on the system’s evolution. Researchers think that there are relationships between history, current, and future. It is more likely a small-world network [31]. Therefore, high-order fuzzy time series models have been developed to describe the fluctuations in a time period and generate forecasting rules that are based on discrete high-order states for future prediction. The most problem of these models is the lack of corresponding rules due to limited training samples. This would significantly reduce the accuracy and credibility of such models. The proposed model summarizes the discrete high-order fluctuation states to truth-membership of upper-trend, falsity-membership of upper-trend, and chaos of trends, and then maps them to the three dimensions of truth-membership, falsity-membership, and indeterminacy-membership of a neutrosophic set. It extends existing high-order fuzzy logical relationships to neutrosophic logical relationships. Therefore, the model can employ NS theory to deal with related issues, such as the comparison of high-order states during the location of rules. Compared with traditional discrete high-order fuzzy logical relationships (FLRs), the proposed to neutrosophic logical relationships (NLRs) have higher generality and can address the problem that is caused by the lack of rules in the forecasting stage.

Inspired by the above research, we propose a prediction model based on high-order fluctuation trends and information entropy. First, the original time series of the stock market is converted into a fluctuation time series, and then the fluctuation time series is blurred into a fuzzy fluctuation time series according to a predefined label. Second, the IE of its historical fluctuations is calculated based on the probability of different states of each current value. Third, the NS is used to represent the current state and to establish a neutrosophic logical relationships. Fourth, the Jaccard similarity measure is used to seek out similar appropriate logical relationship groups and calculate its expected NSs. Finally, we obtain the desired expected NSs, then the final predicted value is calculated through the process of deneutrosophication. For verification, the proposed model is implemented to forecast Taiwan Stock Exchange Capitalization Weighted Stock Index (TAIEX) and Heng Seng Index (HIS). The experimental conclusions indicate that the model has stable prediction ability for different data sets. Simultaneously, comparing the prediction error with other approaches also proves that the model has outstanding prediction accuracy and universality.

The remainder of this article can be summarized as follows: Section 2 introduces some basic concepts of fuzzy fluctuation time series, IE, deneutrosophication of a neutrosophic set, and neutrosophic logical relationships. Section 3 presents a new prediction method that is based on high-order fluctuation trends and NSs. In Section 4, the TAIEX data set (1997–2005) and HIS (1998–2012) are used to predict future values. Section 5 presents the conclusions of this paper and the potential issues for future research.

## 2. Preliminaries

### 2.1. Fuzzy Set (FS)

Fuzzy set theory was proposed by Zadeh [32], and it has been widely applied in several fields. A brief introduction to the basic concepts of fuzzy set follows.

**Definition** **1.** *Denote the universe of discourse as U = {u_1_, u_1_, …, u_n_}. A fuzzy set L* = {*L*_1_, *L*_2_, …, *L_g_*} *in U can be defined by its membership function:*

*L_j_ = f_Lj_(u_1_)/u_1_ + f_Lj_ (u_2_)/u_2_ +…+ f_Lj_ (u_n_)/u_n_ (j = 1, 2, …, g)* (1)

*where f_Lj:_ U → [0, 1] is the membership function of the fuzzy set L_j_, and f_Lj_(u_i_) is the membership degree of u_i_ belonging to L_j_, where f_Lj_(u_i_) ∈ [0, 1] (I = 1, 2, …, n, j = 1, 2, ..., g). The symbol + is not the conventional operation of addition but union.*

Let fuzzy set *L* = {*L*_1_, *L*_2_, …, *L_g_*} be a finite and fully ordered discrete term set, where g is an odd number in real situations with the first (*g* − 1)/2 elements describe the degree of a property and the last (*g* − 1)/2 elements from the opposite description. For example, when *g* = 7, it might represent a set of linguistic variants as: *L* = {*L*_1_, *L*_2_, *L*_3_, *L*_4_, *L*_5_, *L*_6_, *L*_7_} = {very bad, bad, below fair, fair, above fair, good, very good}, and so on. The relationship between the element *L_i_*(*I* = 1, 2, …, *g*) and its subscript *i* is strictly monotonically increasing [33], so the function can be defined, as follows: f: *L_i_* = *f*(*i*). Clearly, the function *f*(*i*) is a strictly monotonically increasing function about a subscript i.

### 2.2. Fuzzy Time Series (FTS)

Fuzzy time series was proposed by Song and Chissom in 1993 [2]. It has been successfully used to solve many practical problems. The brief introduction of basic concepts of fuzzy time series, as follows.

**Definition** **2.** *Let the time series {Y(t)|t = 1, 2, …, T}, a subset of real number, denote the universe of discourse. According to Definition 1, each element in the time series can be fuzzified to a fuzzy set element F(t)(t = 1, 2, …, T), then {F(t)|t = 1, 2, …, T} is called a fuzzy time series defined on the time series {Y(t)|t = 1, 2, …, T}. If there exists a fuzzy relation R(t – p, t), such that*

*F(t) = F(t − p) ◦ R(t − p, t)* (2)

*where ◦ is a max-min composition operator, F(t) is called derived from F(t − p), denoted by the fuzzy logical relationship (FLR) F(t − p) → F(t). F(t – p) and F(t) are called the left-hand side (LHS) and the right-hand side (RHS) of the FLR, respectively. FLRs with the same LHS can be categorized into an ordered fuzzy logical group (FLG).*

If *F*(*t*) is caused by *F*(*t* − 1), *F*(*t* − 2), …, *F*(*t* − *p*), then the p-order FLR can be represented by: *F*(*t* − *p*), *F*(*t* − *p* + 1), …, *F*(*t* – 1) → *F*(*t*)

### 2.3. Fuzzy-Fluctuation Time Series (FFTS)

**Definition** **3.** *Let {X(t)|t = 1, 2, …, T} be a time series, where t is the number of the time series. Let {Y(t)|t = 2,3…T} be a fluctuation time series, where {Y(t) = X(t) − X(t − 1)|t = 2, 3, …, T}. Then the corresponding fuzzy time series {Q(t)|t = 2, 3, …, T} is called a fuzzy-fluctuation time series (FFTS).*

**Definition** **4.** *Let Q(t)* (*t* = *m* + 1, *m* + 2, …, *T*, *m* ≥ 1) *be a FFTS. If Q(t) is caused by Q(t − 1), Q(t − 2), …, Q(t − m), then the fuzzy-fluctuation logical relationship is represented by:*

*Q*(*t* − 1), *Q*(*t* − 2), …, *Q*(*t* − *m*) → *Q*(*t*) (3)

*and it is called the m-order fuzzy-fluctuation logical relationship (FFLR) of the fuzzy-fluctuation time series, where Q(t − 1), Q(t − 2), …, Q(t − m) is called the left-hand side (LHS) and Q(t) is called the right-hand side (RHS) of the FFLR, and Q(k)(k = t, t − 1,t − 2,…, t − m) ∈ L.*

### 2.4. Information Entropy

Information entropy (IE) [12] was firstly defined by Shannon in 1948 as a measure of event uncertainty. Shannon suggested that the smaller the possibility of an incident, the greater the amount of information that it contains. Conversely, the greater the likelihood of an event, the smaller the amount of information. So, the amount of information can be expressed as a function of the probability of occurrence of an event.

**Definition** **5.** (4) $$E=-\overset{N}{\underset{i=1}{\sum }}p\left(x_{i}\right)log_{2}\left(p\left(x_{i}\right)\right)$$

*where p(x_i_) represents the probability of occurrence of the ith event. In addition, the information entropy must satisfy the following conditions:*$\sum_{i=1}^{N}p\left(x_{i}\right)=1,\;0<p\left(x_{i}\right)<1$*and non-negativity:*E≥0.

### 2.5. Neutrosophic Set (NS)

Neutrosophic set (NS) was proposed by Smarandache [20]. It has been widely used to describe complex phenomena.

**Definition** **6.** *Let X be a space of points (objects), with a generic element in X denoted by x. A neutrosophic set A in X is characterized by a truth-membership function T_A_(x), a indeterminacy-membership function I_A_(x), and a falsity-membership function F_A_(x). The functions T_A_(x), I_A_(x), and F_A_(x) are real standard or nonstandard subsets of ]0^−^,1^+^[. There is no restriction on the sum of T_A_(x), I_A_(x), and F_A_(x).*

### 2.6. Neutrosophic Logical Relationship (NLR)

**Definition** **7.** *Let i be the subscript of a fuzzy set element L_i_(i = 1, 2, …, g), A(t) be the LHS of an m-order FFLR* Q(t−1),Q(t−2),…,Q(t−m)→Q(t) *, P^i^_A(t)_ be the probabilities of corresponding L_i_(i = 1, 2, …, g) in A(t). P^i^_A(t)_ can be generated by:*

(5) $$P_{A\left(t\right)}^{i}=\frac{\sum_{j=1}^{m}w_{i,j}}{m}\;\;\;\;\;i=1,2,\ldots ,g$$

*where $w_{i,j}=1$ if Q(t − j) = L_i_ and 0 otherwise.*

Let *A(t)* be the LHS of a *m*-order FFLR Q(t−1),Q(t−2),…,Q(t−m)→Q(t), *E*_*A*(*t*)_ be the entropy of *A*(*t*), *P*^i^_*A*(*t*)_ be the probabilities of corresponding *L_i_*(*i* = 1, 2, …, *g*) in *A*(*t*). *A*(*t*) can be represented by a neutrosphic set *N*_*A*(*t*)_, with the truth-membership function $T_{N_{A\left(t\right)}}$, indeterminacy-membership $I_{N_{A\left(t\right)}}$, and falsity-membership function $F_{N_{A\left(t\right)}}$ defined by:

(6) $$\{\begin{array}{l} T_{N_{A\left(t\right)}}=\sum_{i=1}^{\frac{g-1}{2}}\alpha_{i}P_{A\left(t\right)}^{i}\\ I_{N_{A\left(t\right)}}=E_{A\left(t\right)}\\ F_{N_{A\left(t\right)}}=\sum_{i=\frac{g-1}{2}+1}^{g}\beta_{i}P_{A\left(t\right)}^{i} \end{array}$$

where $\alpha_{i},\beta_{i}$ are the weights of *L_i_*(*i* = 1, 2, …, *g*) in terms of their contribution to represent the corresponding characteristics, $\sum_{i=1}^{\left(g-1\right)/2}\alpha_{i}$ = 1, $\sum_{i=\frac{g-1}{2}+1}^{g}\beta_{i}=1$.

The same as the definition of FLG, *N*_*A*(*t*)_ is called the left-hand side (LHS) of a neutrosophic logical relationship (NLR). The similar LHSs of NLRs can be categorized and group their RHSs of corresponding FFLRs into an ordered fuzzy logical group (FLG) *B_A(t)_*, which can also be represented by a neutrosophic set *N*′_*A*(*t*)_, referring to the above method.

(7) $$\{\begin{array}{l} T_{N_{A\left(t\right)}^{\prime }}=\sum_{i=1}^{\left(g-1\right)/2}\alpha_{i}P_{B_{A\left(t\right)}}^{i}\\ I_{N_{A\left(t\right)}^{\prime }}=E_{B_{A\left(t\right)}}\\ F_{N_{A\left(t\right)}^{\prime }}=\sum_{i=\frac{g-1}{2}+1}^{g}\beta_{i}P_{B_{A\left(t\right)}}^{i} \end{array}$$

where $P_{B_{A\left(t\right)}}^{i}$ represents the probability of corresponding *L_i_*(*i* = 1, 2, …, *g)* in *B_A(t)_*.

Thus, a neutrosophic logical relationship (NLR) can be defined as:$N_{A\left(t\right)}\to N_{A\left(t\right)}^{\prime }$.

**Definition** **8.** *Let N_A(t1)_ and N_A(t2)_ be two NSs. The Jaccard similarity [34] between N_A(t1)_ and N_A(t2)_ in vector space can be expressed as follows:*

(8) $$\begin{array}{l} J\left(N_{A\left(t1\right)},N_{A\left(t1\right)}\right)\\ =\frac{T_{N_{A\left(t1\right)}}T_{N_{A\left(t2\right)}}+I_{N_{A\left(t1\right)}}I_{N_{A\left(t2\right)}}+F_{N_{A\left(t1\right)}}F_{N_{A\left(t2\right)}}}{{\left(T_{N_{A\left(t1\right)}}\right)}^{2}+{\left(I_{N_{A\left(t1\right)}}\right)}^{2}+{\left(F_{N_{A\left(t1\right)}}\right)}^{2}+{\left(T_{N_{A\left(t2\right)}}\right)}^{2}+{\left(I_{N_{A\left(t2\right)}}\right)}^{2}+{\left(F_{N_{A\left(t2\right)}}\right)}^{2}-\left(T_{N_{A\left(t1\right)}}T_{N_{A\left(t2\right)}}+I_{N_{A\left(t1\right)}}I_{N_{A\left(t2\right)}}+F_{N_{A\left(t1\right)}}F_{N_{A\left(t2\right)}}\right)} \end{array}$$

### 2.7. Deneutrosophication of a Neutrosphic Set

**Definition** **9.** *Deneutrosophication of a neutrosophic fluctuation set refers to converting a neutrosophic set x into a fuzzy set y by the following function according to Ali et al. [35]:*

f(T(x),I(x),F(x)):([0,1],[0,1],[0,1])→[0,1]

*Specifically, in this paper, our equation for deneutrosophication is as follows:*

(9) y=γT(x)+θI(x)+δF(x)

## 3. Proposed Model Based on High-Order Fluctuation Trends and Information Entropy

**Figure 1:**
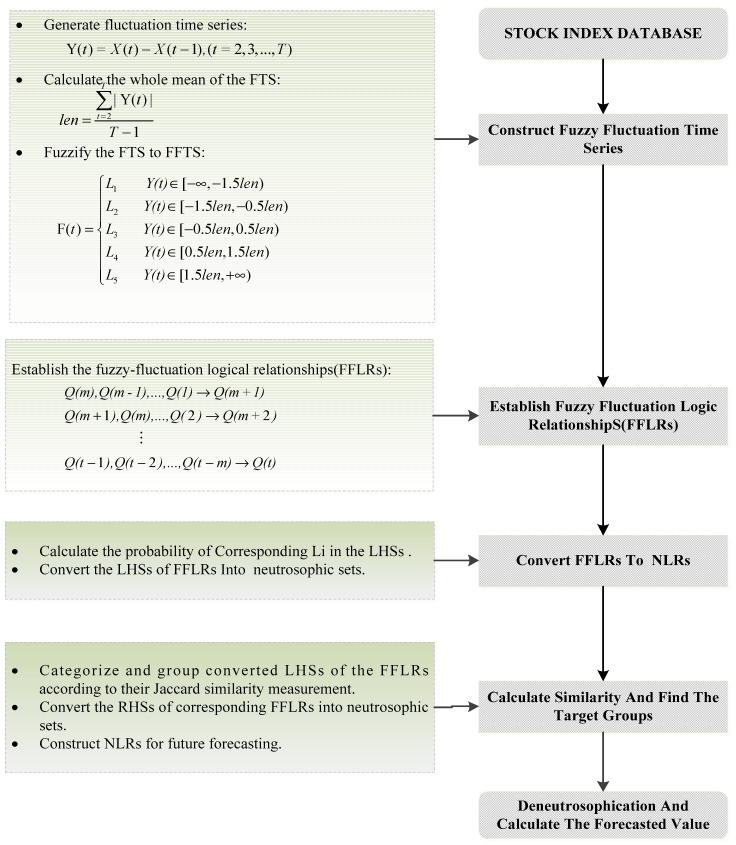
Flow chart of prediction model based on high-order Fuzzy-Fluctuation time series (FFTS) and information entropy (IE).

This paper presents a prediction model that is based on high-order fluctuation trends and information entropy. Different from existing high-order fuzzy time series forecasting models, the proposed model summarizes the discrete high-order fluctuation states into truth-membership of upper-trend, falsity-membership of upper-trend, and chaos of trends. It coincides with the definition of neutrosophic set, which employs the three dimensions of truth-membership, falsity-membership, and indeterminacy-membership to describe a characteristic. Based on the NS expression of high-order fluctuation states, the NS theory can be used to deal with related issues, such as the comparison of high-order states during the location of rules. The most significant contribution of this model is that it extends existing high-order fuzzy logical relationships to neutrosophic logical relationships and it employs information entropy to represent the indeterminacy of fluctuation trends. When compared with traditional discrete high-order FLRs, the proposed NLRs have higher generality and can handle the problem caused by the lack of rules in the forecasting stage. The detailed steps are shown as follow steps and in Figure 1.

Step 1 Construct FFTS for the historical training data.

For each element *X*(*t*)(*t* = 1, 2, …, *T*) in the historical training time series, its fluctuation trend is determined by *Y*(*t*) = *X*(*t*) − *X*(*t* − 1) (*t* = 2, 3, …, *T*). *Y*(*t*) can be fuzzified into a linguistic set {*L*_1_, *L*_2_, *L*_3_, *L*_4_, *L*_5_} = {*down*, *slightly down*, *equal*, *slightly up*, *up}.* Let *len* be the whole mean of all the elements in the fluctuation time series *Y(t)(t =* 2, 3, …, *T)*, and we define intervals $u_{1}=\left[-\infty ,-1.5len\right]$, $u_{2}=\left[-1.5len,-0.5len\right]$, $u_{3}=\left[-0.5len,0.5len\right]$, $u_{4}=\left[0.5len,1.5len\right]$, and $u_{5}=\left[1.5len,+\infty \right]$. Then, *Y(t)(t =* 2, 3, …, *T)* can be fuzzified into a fuzzy fluctuation time series *Q(t)(t* = 2, 3, …, *T)*.

Step 2 Establish *m*-order FFLRs for the training data set

According to Definition 4, each *Q(t) (t > m)* in the training set can be represented by its previous *m* days’ fuzzy-fluctuation numbers. Then, the *m*-order FFLRs of the prediction model can be established.

Step 3 Calculate fluctuation information entropy

According to the Definition 5, the information entropy of the *m*-order fluctuation can be separately calculated. Among them, *p*(*x*_1_), *p*(*x*_2_), *p*(*x*_3_), *p*(*x*_4_), and *p*(*x*_5_), respectively, indicate the probability of occurrence of the linguistic variants *L*_1_, *L*_2_, *L*_3_, *L*_4_, and *L*_5_ in the LHS. Then, the information entropy of the *m*-order FFLRs can be obtained according to Equation (4), which will be the element of the indeterminacy membership of the NSs.

Step 4 Convert the LHSs of FFLRs to NSs

Calculate the probability of corresponding *L_i_*(*i* = 1, 2, …, 5) in the LHSs of training data set, according to Equation (5). Combined with the information entropy of the *m*-order FFLRs obtained in the previous step, convert the left-hands of FFLRs into neutrosophic sets according to Equation (6).

Step 5 Grouping and optimization

Applying the Jaccard similarity measure method, categorize and group the converted LHSs of FFLRs according their similarities. Convert the RHSs of corresponding FFLRs into neutrosophic sets according to Equation (7). NLRs are obtained from the training data sets for future forecasting.

Step 6 Forecast test time series

The Jaccard similarity measurement method can be used to find the most appropriate NLR for each test data set. According to Definition 9, the RHS of the NLR can be converted to an expectation fuzzy value *Y*(*i* + 1). Then, calculate the real number of the fluctuation by: F’(i+1)=Y’(i+1)×len. Finally, the predicted value can be obtained from the actual value of the previous day *X(i)* and the predicted fluctuation value *F^’^(i + 1)*: X’(i+1)=X(i)+F’(i+1).

## 4. Empirical Analysis

### 4.1. Forecasting Taiwan Stock Exchange Capitalization Weighted Stock Index (TAIEX)

The Taiwan stock market has always been the focus of research in the field of stock market forecasting. The TAIEX is an indicator of the change in the value of Taiwan’s overall market stocks, which is seen as a window to the Taiwanese economy. In addition, a large number of studies have used TAIEX data as an example to illustrate their proposed prediction methods [36,37,38,39,40,41]. To facilitate a comparison with the accuracy of these models, we also used the TAIEX data set to illustrate our proposed method. Specifically, while referring to the general practice of the above studies, the forecasting process in this section is based on TAIEX’s 1999 data. Similarly, we also selected data from January 1999 to October 1999 as a training set, and selected from November to December 1999 as a test data set.

Step 1 Construct FFTS for the historical training data

The fluctuation trend is constructed based on the elements of the historical training data set. Then, while using the overall mean of the number of fluctuations in the training data set, the fluctuation trend is blurred to FFTS, e.g., the average value of the training data set for TAIEX. Therefore, *len* is 85. Then, *X*(1) = 6152.43, *X*(2) = 6199.91, and *Y*(2) = *X*(2) − *X*(1) = 47.48. Further, *F*(2) = *L*_4_, means slightly up. Thus, the training set can be converted to fuzzy fluctuation set.

Step 2 Establish *m*-order FFLRs for the training data set

Different orders directly affect the effect of the prediction. Here, we only need to explain the process, so we take the 9th-order as an example. Then, according to Definition 4, the 9th-order FFLRs of the prediction model were established.

Step 3 Calculating fluctuation information entropy

According to Equation (4), the information entropy of the 9th-order fluctuation time series can be calculated separately. For example, the 9th-order FFLRs on November 1, 1999 can be represented by (*L*_3_, *L*_3_, *L*_2_, *L*_4_, *L*_3_, *L*_3_, *L*_3_, *L*_3_, *L*_5_), which can be calculated as *p*(*x*_1_) = 0, *p*(*x*_2_) = 1/9, *p*(*x*_3_) = 6/9, *p*(*x*_4_) = 1/9, and *p*(*x*_5_) = 1/9. Therefore, its information entropy is 1.4466, which can be normalized to 0.5306.

Step 4 Convert the LHSs of FFLRs to NSs

Calculate the probability of corresponding *L_i_*(*i* = 1, 2, …, 5) in the LHSs of the training data set and then convert the left-hands of FFLRs into neutrosophic sets according to Equation (6).

Step 5 Grouping and optimization

**Figure 2:**
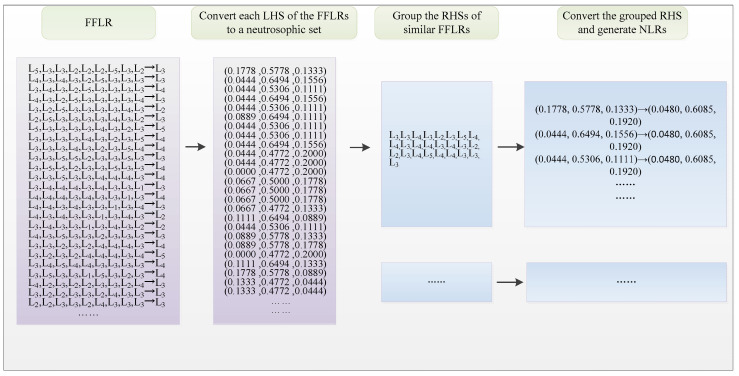
Conversion and group process of fuzzy-fluctuation logical relationship (FFLRs).

Firstly, apply the Jaccard similarity measure method to categorize and group the converted LHSs of FFLRs. In this example, the threshold similarity value is set to 0.94. Then, convert the RHSs of the corresponding FFLRs into neutrosophic sets according to Equation (7). The conversion and grouping process is shown in Figure 2.

Step 6 Forecast test time series

For each test data point, calculate its 9th-order historical fluctuation states and convert them to a neutrosophic set. Locate the NLR with the highest similarity. Then, the corresponding RHS of the NLR is the forecasting neutrosophic value. For example, we chose 1 November 1999 as an example of the test data. The LHS of its fluctuation states is (*L*_3_, *L*_3_, *L*_4_, *L*_3_, *L*_2_, *L*_3_, *L*_5_, *L*_4_, *L*_4_, *L*_3_, *L*_4_, *L*_4_, *L*_3_, *L*_4_, *L*_3_, *L*_2_, *L*_2_, *L*_3_, *L*_4_, *L*_5_, *L*_4_, *L*_4_, *L*_3_, *L*_3_, *L*_3_), which can be represented by a neutrosophic set (0.0480, 0.6085, 0.1920). According to the similarity comparison, the most appropriate NLR for forecasting is (0.0444, 0.5306, 0.1111) → (0.0480, 0.6085, 0.1920).

Then, calculate the predicted fuzzy fluctuation:

Y’(i+1)=(−0.0480)+0.1920=0.144

Calculate the real number of the fluctuation:

F’(i+1)=Y’(i+1)×len=0.144×85=12.24

Finally, The predicted value can be obtained from the actual value of the previous day and the predicted fluctuation value:

X’(i+1)=X(i)+F’(i+1)=7854.85+12.24=7867.09

**Figure 3:**
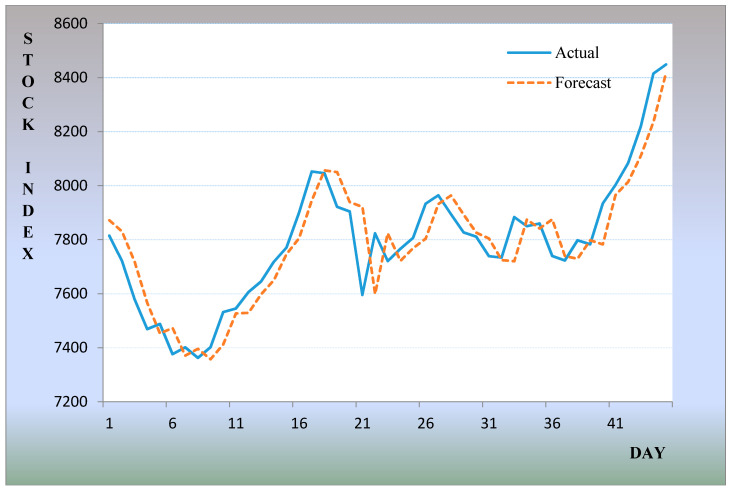
The stock market fluctuation of predicted and actual values for November to December 1999.

According to the above steps, we can obtain other prediction results that are shown in Table 1 and Figure 3.

In experimental analysis, some of the methods are used to measure the accuracy of prediction in order to quantify the effect of model prediction. These methods are mainly used in the prediction field, including the mean percentage error (MPE), the mean square error (MSE), and the root mean squared error (RMSE). Since there is no significant difference in the effect of different error calculation methods, RMSE was chosen as the main formula for error calculation.

(10) $$RMSE=\sqrt{\frac{\sum_{t=1}^{n}{\left(forecast\left(t\right)-actual\left(t\right)\right)}^{2}}{n}}$$

Since different orders affect the prediction effect, in order to reduce the experimental error and improve the accuracy of the prediction, it was necessary to select the optimal order. Experimental analysis showed that when the order was nine, the predictability of the model is better. Table 2 shows the experimental errors for different years under different orders.

It is important and necessary to accurately predict the trend of fluctuations. Some of the most recent articles have proposed many excellent prediction methods. Therefore, by comparing our method with the previous methods, the advantages and disadvantages of our model can be verified.

Table 3 shows the prediction errors for different methods from 1997 to 2005. By comparing with the latest research, we found that the overall prediction effect of this method was excellent. For example, the average error of Guan et al.’s model [40] is 94.5, the average error of Cheng et al.’s model [41] is 102.4, and the error of the proposed model is 92.16. Upon further analysis, from the annual prediction error results, we can see that this method can effectively predict the trend from 1997 to 2005, which is more universal.

### 4.2. Forecasting Hong Kong Heng Seng Index (HIS)

**Figure 4:**
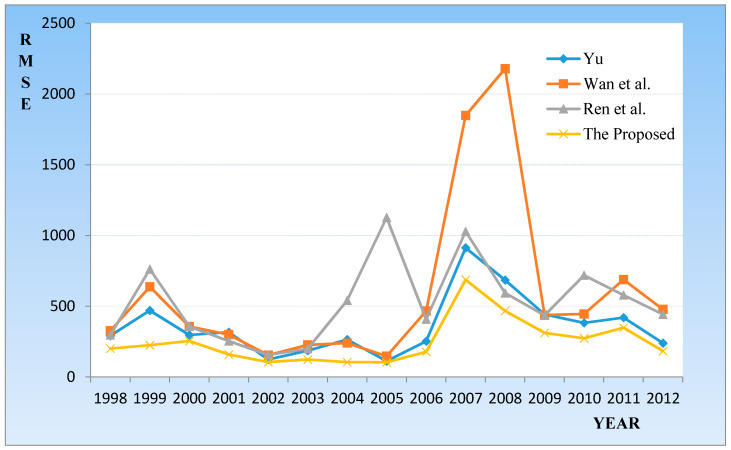
Root mean squared errors (RMSEs) of forecast errors for the Heng Seng Index (HIS) from 1998 to 2012.

HIS is one of the representative indices in Asia. It is not only an important indicator of the Hong Kong stock market price, but is also a stock price index that reflects the most influential trends in the Hong Kong stock market prices. By comparing several authoritative prediction methods, we attempted to verify the universality of the model in other stock markets. Table 4 and Figure 4 compare the different prediction methods from 1998 to 2012.

As shown in Table 4, the average prediction error of the Yu [42] method is 359.66, the Wan [43] method is 395.26, and the Ren [44] method is 526.46. The average prediction error of proposed method is 248.23, which is the smallest error. In addition, it can be seen from Figure 4 that the prediction result of the method is more stable and the prediction effect is more prominent.

## 5. Conclusions

This paper proposed a prediction model that is based on logical rules abstracted from historical dynamic fluctuation trends and the corresponding inconsistencies. During the logical rule training stage, the two dimensions of truth-membership and false-membership of neutrosophic sets were mapped to the dynamic trend states of up and down, respectively. Information entropy was employed to quantify the inconsistency of a period of history and was mapped to the indeterminacy membership of the neutrosophic sets. In the forecasting stage, the similarities among neutrosophic sets were employed to locate the most similar left side of a logical relationship. Therefore, the two characteristics of fluctuation trends and inconsistency assisted with future forecasting. The proposed model was implemented to forecast TAIEX and HIS. The experimental results showed that the model has stable prediction ability for different data sets. Simultaneously, comparing the prediction error with other approaches also proved that our model has outstanding prediction accuracy and universality. This study used two datasets, TAIEX and HIS. Hence, the next study requires data from other stock markets to further validate the model. This study discussed high-order FFTS to characterize the historical fluctuation trends and high-order information fluctuation entropy to measure the inconsistency of historical fluctuations. While considering the relationships among high-order history, current and future, it is likely to use network theory to handle such forecasting problems. In future research, flexible network models should be constructed and more network approaches should be introduced to this area.

## Figures and Tables

**Table 1:** Predicted and actual Taiwan Stock Exchange Capitalization Weighted Stock Index (TAIEX) values from 1 November 1999 to 30 December 1999.

Date (DD/MM/YYYY)	Actual	Forecast	(Forecast − Actual)^2^	Date (DD/MM/YYYY)	Actual	Forecast	(Forecast − Actual)^2^
1/11/1999	7814.89	7867.09	2724.84	1/12/1999	7766.20	7723.08	1859.33
2/11/1999	7721.59	7826.66	11,039.54	2/12/1999	7806.26	7768.72	1408.89
3/11/1999	7580.09	7723.14	20,462.00	3/12/1999	7933.17	7807.11	15,891.12
4/11/1999	7469.23	7577.85	11,798.99	4/12/1999	7964.49	7932.85	1001.33
5/11/1999	7488.26	7466.21	486.03	6/12/1999	7894.46	7964.17	4859.38
6/11/1999	7376.56	7485.29	11,822.59	7/12/1999	7827.05	7893.82	4458.83
8/11/1999	7401.49	7370.82	940.50	8/12/1999	7811.02	7825.78	217.71
9/11/1999	7362.69	7395.82	1097.82	9/12/1999	7738.84	7810.16	5086.43
10/11/1999	7401.81	7357.09	1999.66	10/12/1999	7733.77	7736.72	8.67
11/11/1999	7532.22	7404.90	16,210.15	13/12/1999	7883.61	7734.83	22,134.74
15/11/1999	7545.03	7526.69	336.36	14/12/1999	7850.14	7882.73	1062.35
16/11/1999	7606.20	7541.78	4149.94	15/12/1999	7859.89	7849.27	112.73
17/11/1999	7645.78	7606.20	1566.58	16/12/1999	7739.76	7868.73	16,633.26
18/11/1999	7718.06	7653.40	4180.83	17/12/1999	7723.22	7747.76	602.21
19/11/1999	7770.81	7719.84	2598.34	18/12/1999	7797.87	7730.15	4586.55
20/11/1999	7900.34	7784.54	13,409.46	20/12/1999	7782.94	7799.05	259.55
22/11/1999	8052.31	7919.68	17,589.44	21/12/1999	7934.26	7784.10	22,546.71
23/11/1999	8046.19	8058.99	163.80	22/12/1999	8002.76	7938.25	4161.84
24/11/1999	7921.85	8052.64	17,105.57	23/12/1999	8083.49	8002.58	6546.57
25/11/1999	7904.53	7925.99	460.58	24/12/1999	8219.45	8084.73	18,148.43
26/11/1999	7595.44	7908.62	98,080.84	27/12/1999	8415.07	8224.42	36,348.84
29/11/1999	7823.90	7597.74	51,147.40	28/12/1999	8448.84	8418.62	913.11
30/11/1999	7720.87	7823.13	10,456.55	Root Mean Square Error(RMSE)			102.05

**Table 2:** Comparison of forecasting errors for different *m*-orders.

	*m*								
	6	7	8	9	10	11	12	13	14
RMSE	102.76	102.52	102.2	102.05	102.61	102.89	102.9	102.88	103.4

**Table 3:** Performance comparison of prediction root mean squared error (RMSE) with other models for TAIEX from 1997 to 2005.

Method	RMSE									
	1997	1998	1999	2000	2001	2002	2003	2004	2005	Average
Chen and Chang [36]	N	N	123.64	131.1	115.08	73.06	66.36	60.48	N	94.95
Chen and Chen [37]	140.86	144.13	119.32	129.87	123.12	71.01	65.14	61.94	N	106.92
Chen et al. [38]	138.41	113.88	102.34	131.25	113.62	65.77	52.23	56.16	N	96.71
Cheng et al. [39]	N	N	100.74	125.62	113.04	62.94	51.46	54.24	N	84.67
Guan et al. [40]	141.89	119.85	99.03	128.62	125.64	66.29	53.2	56.11	55.83	94.05
Cheng et al. [41]	N	120.8	110.7	150.6	113.2	66.0	53.1	58.6	53.5	102.4
Our model	140.33	114.35	102.05	129.97	113.32	66.26	54.66	55.19	53.33	92.16

**Table 4:** Performance comparison of prediction RMSE with other models for the Hong Kong Heng Seng Index (HIS) from 1998 to 2012.

Method	1998	1999	2000	2001	2002	2003	2004	2005	2006	2007	2008	2009	2010	2011	2012	Average
Yu [42]	291.4	469.6	297.05	316.85	123.7	186.16	264.34	112.4	252.44	912.67	684.9	442.64	382.06	419.67	239.11	359.66
Wan et al. [43]	326.62	637.1	356.7	299.43	155.09	226.38	239.63	147.2	466.24	1847.8	2179	437.24	445.41	688.04	477.34	595.26
Ren et al. [44]	296.67	761.9	356.81	254.07	155.4	199.58	540.19	1127	407.89	1028.7	593.8	435.18	718.33	578.7	442.44	526.46
Our model	200.72	224.81	254.56	158.88	105.53	122.99	104.51	103.66	177.49	686.79	466.81	311.76	273.49	348.57	182.85	248.23
